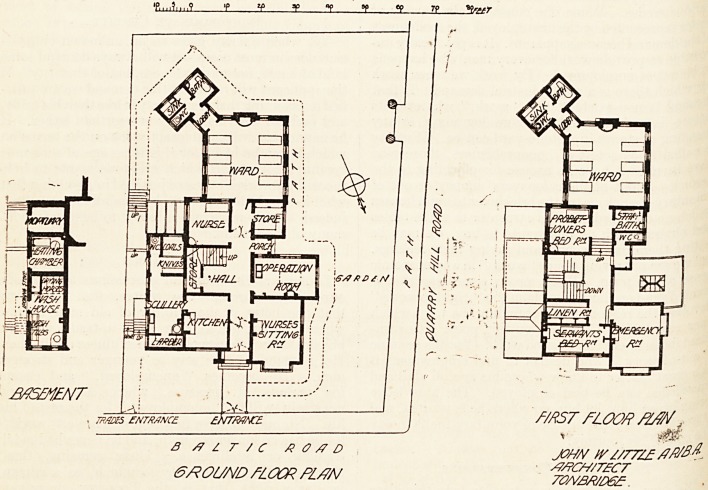# Tonbridge Cottage Hospital

**Published:** 1908-02-08

**Authors:** 


### TONBRIDGE COTTAGE HOSPITAL.

The Queen Victoria Cottage Hospital, Tonbridge, was erected by public subscription throughout the neighbourhood, to commemorate the sixtieth year of her late Majesty's reign. Opinion being divided as to the advisability of opening a cottage hospital within six miles of the large hospital at Tunbridge Wells, only sufficient money was obtained at first to admit of a very small building being erected. The committee, however, felt certain of the need for a hospital in the town, and wisely determined to build with a view to subsequent extension.

A site on the hill overlooking the town having been given by Messrs. Catchpole, Powell, and Potter, the portion which was to be the administration block of the completed hospital was erected at a cost of £1,082, and opened in February 1901. The room destined to become the nurses' sitting room, on the ground floor, served as a male ward; the room over this was used as a female ward; and three patients could be accommodated in each. Within a year the medical officers asked for a small operating room, and this was added at a cost of £151 in 1902.

In 1906 a determined effort was made by the committee to complete the hospital, the need and use of it being by that time well proved. Accordingly plans were made for a twelve-bed hospital, estimates were obtained, and the greater part of the money was collected. The hospital in its complete form was opened for use in February 1907. The additions cost £1,935, bringing the total cost to £3,168.

The hospital, constructed of red brick and tile, stands on rising ground, 70 feet above the rest of the town. Owing to the conformation of the hillside, the main front had of necessity to face west. The male ward, 24 by 20 feet and 12 feet high, is situated on the ground floor. It contains six beds, each having a window on both sides, and it is warmed by radiators and a fireplace. The ward is well lighted and there is ample cross ventilation. The walls and ceiling are finished in Keene's cement, and painted in green; the flooring is of Eubœolith on concrete; while the corners are everywhere rounded. The ward communicates by means of a cross ventilated passage with a sanitary tower, containing w.c., sink room, and bath room.

The female ward on the floor above is precisely similar in all details. Next the male ward is the sister's bedroom; next the female ward is the probationer's bedroom; in each case there is an inspection window commanding the ward.

The operating theatre on the ground floor is quite sufficient in size, and meets the requirements of aseptic surgery. During the daytime it is well lighted by north and west windows and by a large skylight The walls are of painted uralite, the floor is of terrazzo, and all angles are rounded. The hospital is supplied throughout with electric light, and the theatre contains a particularly complete installation.

The hall staircase, of ample width and easy size, has large windows on both sides, and thus aërially disconnects the wards from the administrative department. The arrangements for sanitation and heating seem to be most efficient. The sanitary fittings were supplied by Messrs. Doulton, and the equipment throughout has been of the best material obtainable. The first part of the hospital was built by H. C. Jarvis, the second part by Beale and Son. The honorary architect is Mr. John W. Little, of Tonbridge.

The construction and equipment of this cottage hospital appear to give complete satisfaction to its medical officers, whose wishes have been considered in planning every detail. Great pains have clearly been taken to meet as fully as possible the demands of modern medical science; while from the patient's standpoint the wards are bright and comfortable and airy.

## Figures and Tables

**Figure f1:**